# Special Issue “Innovative Strategies to Target Metabolism, Inflammation, and Oxidative Stress in Human Diseases”

**DOI:** 10.3390/ijms27041888

**Published:** 2026-02-16

**Authors:** Kota V. Ramana

**Affiliations:** Department of Biomedical Sciences, Noorda College of Osteopathic Medicine, Provo, UT 84606, USA; karamana@noordacom.org

Recent studies suggest that oxidative stress, inflammation, and metabolism are interconnected processes involved in the pathophysiology of human diseases and health. Generally, in medical practice, treatment is based on organ-specific defects. For example, pancreatic damage and dysfunction in diabetes, the brain in neurodegenerative diseases, and the body’s immune system in autoimmune diseases. Furthermore, some bacterial and viral infections target specific organs and cause organ toxicity, leading to morbidity and mortality. However, to control chronic diseases, multipronged therapeutic approaches are beneficial. Most human diseases result from impaired crosstalk among multiple pathways and multiple organs [1,2]. Dysregulation of a single pathway can disrupt homeostasis and destabilize multiple interlinked pathways, leading to cellular toxicity, tissue damage, and dysfunction, and eventually to disease pathology. Understanding the key pathways mediated by oxidative stress, inflammation, and metabolism is necessary for a better understanding of disease pathology and treatment.

This special issue is dedicated to understanding the interplay between metabolic imbalance, chronic inflammation, and oxidative stress in the pathophysiology of human diseases. It is well known that exposure of bodies to environmental pollutants, allergens, infectious agents and xenobiotics could increase oxidative stress by generating the various free radical oxygen species (ROS) such as superoxide (O∙^−2^), hydroxyl (OH∙), peroxyl radical (ROO∙), and nitrogen monoxide (NO∙) that exceeds the capacity of the antioxidant defense system; this is mainly carried out by specific enzymes such as superoxide dismutase, catalase, and heme oxygenase. When the antioxidant system is compromised, it leads to redox imbalance and alters oxidative, immune, and metabolic pathways, resulting in tissue damage and dysfunction. In addition, ROS can cause lipid peroxidation and generate various toxic lipid aldehydes such as hydroxynonenal, malondialdehyde, and acrolein [3,4]. These, along with free radicals, cause oxidative damage to DNA, proteins, and lipids, compromising cellular integrity. Furthermore, they can cause mitochondrial dysfunction and impair energy production, promote apoptosis, and alter the expression of redox-sensitive transcription factors. Similarly, oxidative damage alters gene expression via transcription factors, which could contribute to long-term inflammatory and metabolic reprogramming, leading to various clinical symptoms. Thus, oxidative stress is now well recognized as an early pathogenic driver in several human diseases, including neurodegenerative disorders, cardiovascular disease, cancer, and diabetes.

Generally, inflammation is an essential protective response in our bodies that helps get rid of pathogens and repair tissue injury. Although acute inflammation is tightly regulated and self-resolving, chronic inflammation is maladaptive and contributes directly to tissue damage and disease progression. Persistent production of pro-inflammatory cytokines such as TNF-α, IL-6, and IL-1β disrupts tissue homeostasis and impairs regenerative capability in many chronic diseases, including atherosclerosis, type 2 diabetes, cancer, autoimmune disorders, and neurodegenerative diseases.

Several studies suggest that oxidative stress and inflammation are mechanistically interconnected. Oxidative stress activates NF-ĸB-mediated inflammatory signaling pathways and NLRP3-mediated innate immune signaling pathways, which lead to cytokine production [5,6]. Indeed, the recent COVID-19 disease, caused by the coronavirus SARS-CoV-2, has been shown to cause cytokine bursts in many organs [7,8,9]. Furthermore, in addition to producing energy, metabolism is a key regulator of various cellular functions. Similarly, metabolic pathways contribute to the redox balance and immune cell modulation. Impaired metabolic effects due to mitochondrial dysfunction and insulin resistance can lead to oxidative and inflammatory responses. Moreover, the metabolic reprogramming could also determine whether inflammation resolves or persists, providing a direct mechanistic link between metabolism and immune-mediated diseases.

Thus, oxidative stress, inflammation, and metabolism are all required to maintain balance. However, these events need not be in a particular order. For example, metabolic imbalance increases ROS production; oxidative stress activates inflammatory signaling; inflammation further disrupts metabolic regulation; and oxidative stress impairs metabolic pathways. Many human diseases have the most common pathological consequences for disease initiation and progression. In addition, recent studies targeted upstream regulators of oxidative stress, inflammation, and metabolism to prevent disease. Thus, understanding the pathways of oxidative stress, inflammation, and metabolic function can help develop more durable, preventive, and personalized approaches for managing human diseases.

The studies reported in this special issue combine state-of-the-art research articles and comprehensive reviews to explore the roles of oxidative, inflammatory, and metabolic stresses in disease progression and treatment. These articles provide further insight into how the proper balance of oxidative stress, inflammation, and metabolism can mitigate human disease and promote health.

In an interesting research article, Filonov et al. (Contribution 1) investigated the phosphorus-containing analogue of S-adenosyl-L-methionine (SAM), (R,S)-SAM-PH, to evaluate its interactions with various methyltransferases important for epigenetics. They found that (R,S)-SAM-PH donates methyl groups to Dnmt3a and COMT but not to Dnmt1, despite structural similarities between Dnmt1 and Dnmt3a. This selective activity shows that modifying the carboxyl group of SAM can create enzyme-specific methylation tools.

Life-threatening stress can trigger post-traumatic stress disorder (PTSD) with limited treatment options. In a mouse model of PTSD induced by rat exposure, Strekalova et al. (Contribution 2) showed that thiamine and benfotiamine supplementation reduced anxiety-like behavior and locomotor abnormalities. Furthermore, stressed mice showed increased oxidative stress markers and inflammatory gene expression in key brain regions, which were significantly improved by thiamine treatments. These findings suggest that thiamine compounds may be promising antioxidant and anti-inflammatory therapeutic strategies for PTSD.

Another study by Evstifeev et al. (Contribution 3) has shown that macrophages are key immune cells in protecting against tuberculosis (TB), serving as the first line of defense against *Mycobacterium tuberculosis* and determining infection outcomes through their activation state. They found that highly differentiated Th1 CD27^low^ T cells are more effective at activating macrophages than CD27^high^ T cells, and this activation does not depend on reactive nitrogen species. Therefore, CD27^high^ T cells must differentiate into CD27^low^ effector cells to fully stimulate macrophage bacteriostatic functions in TB.

Mitochondrial fusion–fission balance is disrupted in diabetic retinopathy. This is partly due to the hypermethylation and downregulation of the Mfn2 fusion gene. An interesting study by Kowluru and Kumar (Contribution 4) showed that the long noncoding RNA MALAT1 is upregulated under high-glucose conditions and suppresses Mfn2 by promoting promoter DNA hypermethylation in retinal endothelial and Müller cells. Furthermore, MALAT1 silencing has shown to restore Mfn2 activity and mitochondrial function, suggesting a potential therapeutic strategy to prevent diabetic retinopathy. Another research article by Seto et al. (Contribution 5) evaluated the role of valproic acid (VPA) in preventing cisplatin-induced peripheral neuropathy with mechanical allodynia. They have shown that repeated VPA treatment significantly reduced cisplatin-induced allodynia by inhibiting the neurokinin 1 receptor (NK1R) expression in the spinal cord. These results suggest the potential use of VPA for chemotherapy-induced neuropathy.

Another research study by Dyachenko et al. (Contribution 6) examined how imbalances in copper and zinc metabolism are linked to breast cancer. These metals play key roles in redox balance, immune function, and gene regulation. In this study, the authors examined copper and zinc levels in the saliva of breast cancer patients and their relationship with oxidative stress, antioxidant and immune status, and amino acid metabolism. Similarly, an article by Kadoh et al. (Contribution 7) showed that serum amyloid A (SAA) was elevated only in affected pseudoexfoliation syndrome (PE)+ eyes, while IL-8 and ET-1 were increased in both PE+ and clinically unaffected fellow eyes compared to controls. Interestingly, IL-8 was associated with worse visual acuity, whereas ET-1 showed an inverse association with visual acuity. These findings suggest that IL-8 and ET-1 could serve as early biomarkers for preventing glaucomatous damage before PE becomes clinically visible.

This special issue also features six informative review articles. Khan et al. (Contribution 8) discuss the significance of gene- and RNA-based therapies as promising new treatments for diabetes. They discuss recent encouraging preclinical results and ongoing clinical trials. This review further highlights current research gaps and future directions, emphasizing interdisciplinary efforts toward personalized diabetes therapies and regulatory considerations. Meanwhile, Gliozheni et al. (Contribution 9) discuss how traditional foods such as shiitake, turmeric, ginseng, berries, and holy basil act as antioxidants and immune modulators by activating pathways such as Nrf2/Keap1 and shifting Th1/Th2 immune responses. They discuss their anti-inflammatory, antiangiogenic, and gut-microbiota-modulating effects, based on studies from 1994 to 2024. In another article, Folta et al. (Contribution 10) discussed how inflammation plays a central role in neurodegenerative diseases such as Parkinson’s, Alzheimer’s, and multiple sclerosis. They specifically explored how chronic neuroinflammation, driven by activated microglia and astrocytes, contributes to ongoing neuronal injury both in the brain and systemically. This review further discusses how targeting neuroinflammatory pathways may lead to new therapeutic strategies beyond current symptomatic treatments.

It is well known that vitamin B12 is essential for DNA synthesis and cellular metabolism. A review article by Kuldyushev et al. (Contribution 11) highlights recent advances in B12 delivery systems and their role in improving drug delivery. They have also discussed B12’s unique absorption mechanisms, its complex metabolic network, and the health consequences of deficiency in neurological and blood disorders. In another review article, Wen et al. (Contribution 12) discussed how mitochondrial DNA (mtDNA) can act as an immunostimulatory signal released during mitochondrial damage via receptors such as TLR9, cGAS, and NLRP3. They specifically show how exercise influences this process. Interestingly, this article highlights current knowledge gaps and suggests that understanding exercise–mtDNA–immunity interactions could lead to new diagnostic and therapeutic strategies for immune-related diseases. Finally, Srejovic et al. (Contribution 13) examined how VEGF plays a central role in retinal vascular diseases. They discuss the involvement of interconnected signaling pathways in retinal blood vessel development and current therapies.

Recent and currently published studies in this special issue suggest that oxidative stress, inflammation, and metabolism represent interlinked biological processes that maintain cellular equilibrium and disease progression. The impairment of any one process can destabilize others, leading to pathological consequences. Targeting upstream regulators of redox balance, inflammatory signaling, and metabolic pathways offers more potent therapeutic opportunities than treating isolated symptoms. Further, lifestyle and environmental factors play a key role in regulating these processes, and advances in precision medicine and multi-omics technologies will enable the personalized modulation of oxidative, inflammatory, and metabolic pathways. Identification of specific biomarkers common to these three pathways could improve early diagnosis. Ultimately, restoring balance among oxidative stress, inflammation, and metabolism is a fundamental strategy for maintaining human health and preventing disease.
